# Correction: Rigid geometry solves “curse of dimensionality” effects in clustering methods: An application to omics data

**DOI:** 10.1371/journal.pone.0243498

**Published:** 2020-12-03

**Authors:** Shun Adachi

In S1 Appendix the first sentence of the second paragraph is incorrect. The correct S1 Appendix is below.

## Supporting information

S1 Appendix Utilizing a *p*-adic metric embedded in rigid geometry. Appendix indicating calculation of the new metric *v*.
(DOCX)
