# Supplementary material for: Rigid geometry solves “curse of dimensionality” effects in clustering methods: An application to omics data
Source: PLoS One. 2017 Jun 14;12(6):e0179180. doi: 10.1371/journal.pone.0179180 (PMC5470695; doi:10.1371/journal.pone.0179180)
Supplement: S1 Appendix — Appendix indicating calculation of the new metric v. (DOCX) [file pone.0179180.s001.docx]

Utilizing a *p*-adic metric embedded in rigid geometry

We used an analogy (a grounding metaphor) for the biological data space (the base) and the mathematical space (the target). We will not go into detail about of the reverse direction of the analogy, because we still do not understand in detail the mathematical behavior of the target space. We will consider a projection from the base to the target, and we will discuss the theory of the projected data (linking metaphors). The improvement in the metric for the unused values was based on rigid geometry, as follows; also see [1]. The data were arranged according to rank *k* of the unused values *N_k_*, according to a logarithmic approximation:

$$N_{k}=a-b\ln k$$

The *R^2^* values for this approximation were approximately 0.84−0.95 in LC/MS, 0.58-0.61 in *Saccharomyces cerevisiae* expression array and 0.95-0.98 in *Escherichia coli* induction factor. Then,

$$r=\frac{\ln\frac{a}{N_{k}}}{\ln k}$$

was calculated from the logarithmic distribution as a deviation index. After that,

$$|D|=e^{\frac{r}{b}}$$

was calculated as an index of absolute fitness for ln(protein density), as predicted from the logarithmic Boltzmann distribution of the protein signals. The average signal of the sample *E*(*N*) was calculated, and the expected average overall cooperative fitness of each protein was calculated as

$$p={|D|}^{E(N)}$$

for a *p*-adic metric. A Euclidean metric of the complex-valued$s=r+p\sqrt{-1}$, $\sqrt{(r_{2}-r_{1})^{2}+(p_{2}-p_{1})^{2}}$ from any two points $s_{1}=r_{1}+p_{1}\sqrt{-1}$ and $s_{2}=r_{2}+p_{2}\sqrt{-1}$ obviously satisfies necessary conditions for the general metrics introduced in the Introduction. It also fulfills the requirements for a parameter of a high-dimensional theta function that converges absolutely and uniformly on a complex three-dimensional compact subset [1, 2]. To understand this, we can relate the theta function to the upper half-plane $\{\mathbb{H}\ni\forall s | s\in\mathbb{C} \left( \mathfrak{I}\left( s \right)>0 \right)\}$. Let $\frac{1}{\zeta k^{s}}\in\mathbb{C}$, $\mathbb{R}=\left[ \prod\mathbb{C} \right]^{+}=\left\{ s\in\mathbb{C} | s=\bar{s} \right\}$ as in theta series of [2], and let the Hecke ring $\mathbb{R}$ be the Minkowski space, which treats the time dimension different from other three dimensions, *a*, *b* and *k*. Then, the dual space of the Hecke ring $\left\{ \mathbb{R}_{+}^{*} \right| \mathbb{R}_{+}^{*}\ni\forall p\}$ and a set of the natural logarithm of *p* is $\left\{ \mathbb{R}_{\pm}|\mathbb{R}_{\pm}\ni\forall E\left( N \right)\ln|D| \right\}\subseteq\left\{ \mathbb{R}_{b}|\mathbb{R}_{b}\ni\forall b\ln\left| D \right| \right\}$. It is notable that $\left\{ s \mathbb{\in H} \right| \mathbb{H} =\mathbb{R}_{\pm}+\sqrt{-1}\mathbb{R}_{+}^{*}\}$.

Then,

$$\mathbb{H\subseteq C\supseteq R\supseteq}\mathbb{R}_{\pm}\supseteq\mathbb{R}_{+}^{*}$$

is the form we need for a theta function that converges absolutely and uniformly on every compact subset $\mathbb{R}\times\mathbb{R}\times\mathbb{H}$ [2]. We can say that *s* and especially *p* are quasi-compact and quasi-separated, based on the number theory [2].

Finally, a flag manifold with reverse direction of *k* ($\frac{\ln N_{k}}{p}< \frac{\ln N_{k-1}}{p}<\ldots< \frac{\ln N_{1}}{p}$)

$$v=\frac{\ln N_{k}}{\ln p}$$

was calculated, and the set of *v* is a coherent rigid analytic space with a coherent formal scheme $\left\{ \mathbb{S}_{F} \right|\mathbb{S}_{F}\ni\forall\ln N_{k}\}$ divided by a *p*-adic blowup ln *p*, and it is quasi-compact and quasi-separated [3, 4] (note that it must be an adequate formal scheme but it is not necessary for it to be a Noetherian scheme with an ascending/descending chain condition, and thus we are able to include non-Noetherian schemes, such as an irreversible time-asymmetric model). If we assume that $\ln N_{k}$ is a Tate algebra *T_n_* (if we set an observed set of *v* in each protein/gene as *v_1_*, *v_2_*, …*v_n_* and Tate algebra as an ideal in *i*th protein/gene *X_i_* = *p_i_*, $T_{n}(k) := k<X_{1}, \ldots, X_{n}> := \{\sum_{v_{1,\ldots,}v_{n\geq0}} a_{v_{1,\ldots,}v_{n}}{X_{1}}^{v_{1}}\ldots{X_{n}}^{v_{n}}; a_{v_{1,\ldots,}v_{n}}\in\{k\} \mathrm{and}|a_{v_{1,\ldots,}v_{n}}|\to0 for v_{1}+\ldots+v_{n}\to\infty\}$, this is the only necessary assumption with any ideal *I* of *T_n_* being closed and *T_n_/I* being a finite field extension of a ground field [5]), a quotient by an ideal ln *p* is isomorphic to a *k*-Banach algebra, which is an affinoid algebra that is quasi-compact and quasi-separated. Note that *v* becomes affinoid in locally closed immersions when the projections are bijective, neglecting the case *p* = 1 [6, 7] (note that in the original non-Archimedean valuation, it should be –*v*; however, the negative sign is not relevant to its use as a metric). From arithmetic calculations based on the −*v* *N_k_* space, *v* is on an ultrametric space; however, we calculate *v* from *N_k_* and consider it on a Euclidean metric space. Note that from the original unused values, the space is *p*(*I*)-adic, not Euclidean. A Euclidean metric of two independent *v*, *v_1_* and *v_2_*, $\sqrt{(v_{2}-v_{1})^{2}}$ obviously satisfies the four requirements for general metrics. We did not analyze the top *k* = 1 proteins, because calculation by 1/ln *k* could not reach infinity.

For the proof that $\left\{ V \right| V\ni\forall v\}$ obeys rigid geometry, we consider a Schottky-type uniformization of the elliptic curve on the complex plane. Let Λ be a periodic lattice, let the expectation of *N* be *E*(*N*), and let the normalization factor of *P* be *N_k_*/*E*(*N*) = *PD^Nk^*; then

$$\Lambda=2\pi\sqrt{-1}\left( \mathbb{Z}+\tau\mathbb{Z} \right)$$

$$\left( \tau\in\mathbb{H} \right)$$

$$\ln p^{v}=\ln N_{k}\in\mathbb{C}$$

$$\mathbb{C}\underset{\to}{\exp}\mathbb{C}^{\times}\ni N_{k}=p^{v}$$

$\mathbb{C}/\Lambda={\mathbb{C}^{\times}}/{q^{\mathbb{Z}}}=\left\{ \mathbb{S | S}\ni PE\left( N \right)D \text{mod}N_{k}=\frac{PD^{N_{k}}}{\frac{N_{k}}{E\left( N \right)}} \right\}$.

An infinite-dimensional covering of the final equation is a Schottky-type uniformization (c.f. [3]). Note that |*q*|*_p_* < 1. This uniformization is identical to utilizing a geometric prime number theorem *PE*(*N*)*π^G^*(*x*) ~ *PE*(*N*)*e^x^*/*x*, where *π^G^*(*x*) is a prime counting function of the value *x* = *N_k_* [8]. On a Weierstrass elliptic curve, *E*: *y*^2^ = 4*x^3^* – *g_2_x* – *g_3_*, the Tate curve is realizable only if in the following conditions. Remember a Klein’s *j*-invariant [3], which is a modular function of weight 0 for $\mathrm{SL}(2, \mathbb{Z)}$ defined on $\mathbb{H}$. The rational functions of it gives a modular function of any types, because it gives bijection from complex numbers to isomorphic classes of elliptic curves on $\mathbb{C}$ [2]. Tate curve is realizable if

$${\left| j(E \right)|}_{p}=\left| 1728\frac{g_{2}^{3}}{g_{2}^{3}-27g_{3}^{2}} \right|>1$$

which means we assume *g_2_* << 1; if |*g_3_*| >> 1, this would result in a collapse of the Tate system. That is, if the external effects on the *x* variable are too large, the efficiency of the interaction among the *x* that constitute *y^2^* would become unrealizable (the collapse of the Tate system), and this limits the size of system.

Let *M* be a differentiable manifold, let Ω^0^(*M*) be a smooth function space on a rigid analytic space *M*, and let Ω*^i^*(*M*) be a space of the *i*-th differential form. Then, $d^{i}: \Omega^{i}(M)\to\Omega^{i+1}(M)$ represents the exterior derivative, and the elements of Ker *d^i^* and Im *d^i^* (the image of the map *d^i^*) are of closed form and exact form, respectively. Thus, *d^i+1^ d^i^* = 0 and

$$0\to\Omega^{0}\left( M \right)\to\Omega^{1}\left( M \right)\to\Omega^{2}\left( M \right)\to\Omega^{3}\left( M \right)\to\ldots$$

is a crystalline complex, cohomological to a crystalline cohomology [9, 10]. That is,

$$H_{dR}^{i}\left( M \right)=\text{Ker} d_{i}/\text{Im}d_{i-1}$$

is the *i*-th crystalline cohomology group. Therefore *H^i^_dR_* = 0 and “any *i*-th closed form is an exact form” are equivalent. We can take a set in rigid analytic space, mod *N_k_*, as Ω. Please note that if we set *p* as an element of a Coxeter group, an identity element of *p* corresponds to an identity element of a Hecke ring. Thus, *d* = *p*. Furthermore, *i* = *v* is smooth when *p* ≠ 1. Since the exterior derivative of *p* is 0, and *p* is obviously of exact form unless *v* = 0, *H^i^_dR_* = 0. Then, *v* = ln *N*(*t*)/ln *p* (*t* is time) is obviously on the unit polydisc in rigid analytic space, with renders a locally ringed *G*-topologized space with a non-Archimedean field as a sheaf; this ensures a covering by open subspaces isomorphic to affinoids. Shifting –*v* (non-Archimedean valuation for 1/*N_k_* = *p*^-^*^v^*) to the *v* metric (non-Archimedean valuation for *N_k_* = *p^v^*) does not change this property, when considering the 1/*N_k_* space as the basis. In other words, ln *N_k_* is related to the kernel of the present signal space, and ln *p* is related to the kernel of the potential signal space, which is the image of the past signal space. Their quotient, that is *v,* is the image of the potential signal space, which reflects the physiology of the system adapted to the expected environment without noise. Overall, the system described here has a rigid cohomology [11]. For *N_k_/E*(*N*) = *PD^Nk^* and *P* = 1/(*D^a^ζ*(*s*)) (Adachi 2016),

$$v=\frac{N_{k}\ln D-a\ln D-\ln\zeta\left( s \right)+\ln E(N)}{E\left( N \right)\ln\left| D \right|}$$

Overconvergence of *v* is thus achieved due to the cancelling out of high dimensionality in *N* and *a*, and the topological characteristics (*G*-topology) of *v* (quasi-compact and quasi-separated), as discussed above.

**References**

1. Adachi S. Discrimination of domination mode and chaotic mode in species; 2016. Preprint. Available from arXiv:1603.00959v5 [q-bio.PE]. Cited 25 December 2016.
2. Neukirch J. Algebraic number theory. Berlin-Heidelberg-New York: Springer Verlag; 1999.
3. Fujiwara K, Kato F. Rigid geometry and applications. Adv. Stud. Pure Math. 2006; 45:327-386.
4. Kato F. Topological rings in rigid geometry. In: Cluckers R, Nicaise J, Sebag J, editors. Motivic integration and its interactions with model theory and non-Archimedean geometry, Vol. I. London Math. Soc. Lecture Note Ser., 383. Cambridge: Cambridge Univ. Press, 2011. pp. 103-144.
5. Bosch S, Güntzer U, Remmert R. Non-archimedean analysis. Berlin-Heidelberg-New York: Springer-Verlag; 1984.
6. Gerritzen L, Grauert H. Die Azyklizität der affinoiden überdeckungen. In: Spencer DC, Iyanaga S, editors. Global analysis (Papers in honor of Kodaira K). Tokyo: Univ. Tokyo Press, 1969. pp. 159-184.
7. Temkin M. A new proof of the Gerritzen-Grauert theorem. Math. Annal. 2005; 333: 261-269.
8. Chebolu SK, Mináč J. Counting irreducible polynomials over finite fields using the inclusion-exclusion principle. Math. Mag. 2011; 84: 369-371.
9. Grothendieck A. On the de Rham cohomology of algebraic varieties. Institut des Hautes Études Scientifiques. Publications Mathématiques. 1966; 29:95-103 (Letter to Atiyah, 14 October 1963).
10. Grothendieck A. Crystals and the de Rham cohomology of schemes. In: Grothendieck A, Kuiper NH, editors. Dix Exposés sur la Cohomologie des Schémas. Advanced Studies in Pure Mathematics, 3. Amsterdam: North-Holland Publishing Co., 1968. pp. 306-358.
11. Kedlaya KS. *p*-adic cohomology. In: Abramovich D, Bertram, A, Katzarkov L, Pandharipande R, Thaddeus M, editors. Algebraic geometry-Seattle 2005, Part 2. Proc. Sympos. Pure Math., 80. Providence: Amer. Math. Soc., 2009. pp. 667-684.
